# The Impact of Pre-Round Meetings on the Clinical Learning of Nurses and Doctors on Hospital Wards: A Qualitative Study

**DOI:** 10.1177/23779608221094719

**Published:** 2022-04-20

**Authors:** Ole T. Kleiven, Irene Sjursen, Lars Kyte

**Affiliations:** 1 Faculty of Health and Social Sciences, 150769 Western Norway University of Applied Sciences, Førde, Norway

**Keywords:** pre-round meetings, learning, hospital wards, interdisciplinary cooperation, qualitative study

## Abstract

**Introduction:**

The pre-round meeting is an interprofessional gathering conducted in conjunction with the ward round in many hospitals. Here, nurses, doctors and eventually allied health clinicians discuss clinical issues before attending to the patients. This study focused on the learning aspects of the pre-round meeting, and it is, to our knowledge, the first study to explore the impact of pre-round meetings on learning in a clinical setting.

**Objectives:**

To improve our understanding of the impact pre-round meetings has on clinical learning among the nurses and doctors who attend them.

**Method:**

A qualitative study. Focus group interviews were conducted. Participants comprised of 9 doctors and 13 nurses from two different hospitals in Norway. The participants represented both surgical and non-surgical departments

**Results:**

This study showed that the pre-round meeting is an arena with a high learning potential. Learning takes place in the discussion that arises when different professions meet. Both nurses and doctors emphasized that the pre-round meeting is both a conscious learning arena and an arena where learning is a by-product. Several factors interfered with the utilization of its learning potential.

**Conclusion:**

The pre-round meeting is an arena with high learning potential. However various factors can influence this potential. The study highlights the importance of being aware of the learning potential in the pre-round meeting, to achieve higher-level learning objectives. A collaborative environment, continuity, competence, and availability of the staff and structured pre-round meetings are essential elements for achieving higher-level learning objectives.

## Introduction

Interprofessional team meetings are important learning arenas in clinical practice. Learning happens through discussion with other participants in these meetings, and by listening to the other professionals attending them (Nisbet et al., 2015). The pre-round meeting is an interprofessional gathering conducted in conjunction with the ward round in many hospitals. Here, nurses, doctors and eventually allied health clinicians discuss clinical issues before attending to the patients. Ward rounds are an important activity in hospitals (Beigzadeh et al., 2019; Gray et al., 2020; O’Hare, 2008). The purpose of rounds may either be planning patient care, education or a combination of the two (Walton et al., 2016). Often ward rounds are multidisciplinary, where physicians, nurses, and various kinds of allied health clinicians, as pharmacists, occupational therapists, physiotherapists, or social workers are represented (Walton et al., 2016). It is structured in different ways and consists of several segments, including the preparation for the ward round, the ward round itself (where physicians and nurses attend to the patient), and the follow-up period afterwards (Lees, 2013; Royal College of Physicians & Royal College of Nursing, 2012; Willemann et al., 2006). The pre-round meeting is part of the preparation for the ward round.

The ward round offers opportunities for improving patient care and for providing learning opportunities for all attendees (Beigzadeh et al., 2019; Royal College of Physicians & Royal College of Nursing, 2012). Nevertheless, the value of the ward round as a learning arena has been underestimated and rarely investigated (Bell et al., 2016; Laskaratos et al., 2015).

Learning can be classified as formal, non-formal and informal. Formal learning occurs within an organized and structured context, is designed as learning, and is intentional (Commission of The European Communities, 2001; European Commission). It is known to be most effective when it is relevant and well-timed (Eraut, 2007). Non-formal learning occurs in activities not explicitly designed as learning, but the learning is intentional from the learner's point of view. Informal learning results from daily life activities, for example, those related to work, and in most cases, it is non-intentional (Commission of The European Communities, 2001; European Commission).

Collaboration in practice is closely related to learning (Nisbet et al., 2015). Learning in the pre-round meeting may contribute to improvement of health outcomes, as knowledge-building occurs in everyday practice where people meet and discuss (Mylopoulos & Scardamalia, 2008, Nisbet et al., 2015). A discussion before attending to patients may facilitate learning (Claridge, 2011). Learning may also take place through joint decision making (Radomski & Beckett, 2011), something that can improve the quality of care (Kyte et al., 2020). In this paper, we discuss how the pre-round meeting may contribute to the clinical learning of those who attend them.

## Review of Literature

Interprofessional learning refers to enabling the gathering of different professions to share knowledge familiar to the members of one profession, but not to the other profession. Learning together and from each other enhances collaboration among professionals and improves health outcomes of patients (Bell et al., 2016; Health Professions Networks Nursing & Midwifery Human Resources for Health, 2010). Interprofessional learning presupposes an arena where professionals can meet. Research shows that the knowledge gained at interprofessional team meetings may benefit the individual learner and contribute to the collective learning of the healthcare team, thereby improving the treatment and care of patients (Nisbet et al., 2015). Learning occurs while observing something from a different perspective, and nurses and doctors have different approaches to clinical issues (Kyte et al., 2020; Nisbet et al., 2015; Sjursen & Ytrehus, 2020).

The pre-round meeting ensures opportunities to meet, because it is scheduled at a time of day when staff, involved in the treatment and care of patients, can gather to discuss clinical issues. Although different professions participating in the pre-round meeting have different points of view, the goal is to provide the best possible care and treatment for patients. When the group has common goals and makes joint decisions to achieve them, an environment conducive to learning is created (Bell et al., 2016).

To our knowledge learning in pre-round meetings is hardly studied. However, learning in the ward round itself is far more investigated, and several articles discuss ways of improving learning during ward rounds (Gray et al., 2020; Tariq et al., 2021). Though ward rounds may be successful learning arenas, several factors affect the learning environment. Workload, lack of time, number of patients, high patient turnover, frequent interruptions and lack of interest among senior staff members may impede learning during ward rounds (Laskaratos et al., 2015; Seltz et al., 2016). It is a question of how these factors affect learning in the context of the pre-round meeting. This study, therefore, focused on the learning aspects of the pre-round meeting, and it is, to our knowledge, the first study to explore the impact of pre-round meetings on learning.

The aim of this study was to gain a better understanding of the impact of pre-round meetings on learning among the nurses and doctors who attend them.

## Method

### Design

We used a qualitative approach to evaluate the impact of pre-round meetings on learning among nurses and doctors in clinical practice. The analysis was inspired by phenomenology and hermeneutics. As the aim was to obtain an in-depth understanding of different participants’ perceptions and experiences, a qualitative design was more appropriate than a quantitative approach (Hammarberg et al., 2016; Patton, 2015). We chose focus group interviews with nurses and doctors as our approach to obtaining knowledge about how these professionals experience the pre-round meeting as a learning arena. Such an approach is well suited to studying environments in which many people cooperate (Carey, 1995; Malterud, 2017).

### Data Collection, Study Participants and Settings

Participants in the study were 9 doctors and 13 nurses from two different hospitals in Norway. They were recruited by contacting the administration of the health trusts in these two different geographic regions. The recruited doctors (2 women and 7 men) had medical experience ranging from 2 to 40 years, while the nurses (12 women and 1 man) had 0.5 to 30 years of nursing experience. The participants represented both surgical and non-surgical departments (Table 1). The way pre-round meetings were held, varied among departments. Duration varied from 20 min to about 2 h. Generally, they were shorter in surgical and orthopedic wards than in medical wards and in the intensive care unit. In some wards, the meetings were divided into groups. At all departments, nurses, and doctors (either consultants or registrars or both) attended. At most units allied health clinicians and students sometimes attended, the frequency of this varied among wards. One of the participants worked on a unit where they did not have regularly pre-round meetings.

**Table 1.** Overview of the Study Participants.

Focus group	Participant number	Gender (f/m)	Position	Current field of practice
A (Nurses)	1	f	Team nurse	Department of general medicine
	2	m	Nurse	Observation unit
	3	f	Cancer nurse	Cancer ward
	4	f	Lead nurse	Medicine and surgery
	5	f	Assistant ward nurse	Surgical ward
	6	f	Assistant ward nurse	Surgical ward
	7	f	Nurse	Department of general medicine
B (Doctors)	1	m	Consultant	Children's ward
	2	m	Consultant	Neurology ward
	3	m	Consultant	Neurology ward
	4	m	Doctor, specialist in anesthesia	Intensive care/anesthesia/operating theater
C (Nurses)	1	f	Nurse	Neurology ward
	2	f	Nurse	Neurology ward
	3	f	Nurse	Surgical ward
	4	f	Critical care nurse	Intensive care unit
	5	f	Nurse	Orthopedic ward
	6	f	Nurse	Children's ward
D (Doctors)	1	f	Consultant	Department of general medicine
	2	m	Registrar	Orthopedic ward
	3	m	Senior consultant	Orthopedic ward
	4	f	Registrar	Surgical ward
	5	m	Registrar	Department of general medicine

Abbreviations: f, female; m, male.

Focus groups may be performed in different ways. In this study, nurses and doctors were interviewed in separate groups to give each profession space to express their experiences without being influenced by other professions. The interviews were conducted by a moderator and an assistant moderator (Lerdal & Karlsson, 2008). All interviews were moderated by the same researcher (LK), while the other researchers (OTK) and (IS) served as assistant moderator at two interviews each. The interviews were based on guidelines from a semi-structured thematic interview guide, consisting of four main themes:

- The impact of pre-round meetings on the quality of patient treatment and care
- Pre-round meetings as a forum for cooperation
- Pre-round meetings as an opportunity for learning
- Potential to improve pre-round meetings

Data from themes i, ii and iv are presented in a previous article (Kyte et al., 2020). In this article, we focus mainly on theme iii.

The interviews lasted between 1 and 2 h, and all interviews were audio recorded and subsequently transcribed.

### Thematic Analysis

Transcripts of the interviews were analyzed thematically using systematic text condensation, as described by Malterud (Malterud, 2012; Malterud, 2017). First, we read through the transcripts to form an overall impression of the content and to outline the main topics in the interviews. These topics were the foundation for the categories (coding groups). Second, we assigned the units of meaning from the transcripts to these categories. The main category corresponding to theme iii in the interview guide was ‘Learning on the ward’. This category was further divided into the subcategories shown in Table 2. Third, we reduced the contents of the units of meaning in each category to condensates in the first-person statements (‘artificial quotes’), in accordance with the description of the method (Malterud, 2012; Malterud, 2017). The subcategories further evolved as some of the categories emerged during the analysis. We also selected genuine quotes from the transcripts. The analyzed text for each subcategory was synthesized based on the first-person statements (‘artificial quotes’) and the selected genuine quotes (Malterud, 2012; Malterud, 2017). This analyzed text formed the basis of the results reported in the Results section.

**Table 2.** Overview of the Main Category and Subcategories.

Learning on the ward How learning (in the pre-round meeting) takes place and for whom What is being learned Factors affecting learning Learning outside the pre-round meeting

### Ethical Approval and Informed Consent

The participants were informed in writing about the project, and signed consent forms before participation. The information in the letter sent to them stated explicitly that the interviews would be audiotaped, and that participation in the study was optional. The Norwegian Center for Research Data (NSD, no. 33036) approved the study protocol. The regional ethics committee found that the request for approval was not required, and thus, the study could be conducted without it.

## Results

### The Pre-Round Meeting: a Central Arena for Learning

Most of the participants in the study (both nurses and doctors) expressed the view that the pre-round meeting was a central arena for learning. Participants, especially junior doctors and newly qualified nurses highlighted the importance of pre-round meetings as an arena for learning, and a training ground for students. Among various participants in the study the following statements were expressed:

I feel it is an invaluable learning arena for nurses, junior doctors and medical students. (A3)

… purely, ad hoc, the pre-round meeting is probably the most important arena for the clinical training of younger doctors. (D5)

It is part of the job for a nursing student to learn to participate in ward rounds on surgical and internal medicine units. (D3)

The pre-round meetings was not only described as a learning arena for less experienced professionals. It was perceived as a useful learning arena for the more experienced doctors as well, as stated this way:

… it's probably also the case that I, as a specialist, also learn from pre-round meetings, because someone poses challenging questions to me that I have to deal with. (D1)

Several participants, both nurses and doctors, pointed out the high learning potential of pre-round meetings when they are fully utilized:

I would say that's where I have probably learned the most on the unit. (A1)

… it's an arena that in a way gives great opportunities for learning if you use it [the pre-round meeting]. (D4)

### What Makes the Pre-Round Meeting an Essential Learning Arena?

The nurses perceived the pre-round meetings as a conscious learning arena and a forum where learning is a by-product. The medical staff too, described the pre-round meeting as a conscious learning arena, for example, when showing Electrocardiograms (ECGs) and blood tests, and commenting on clinical findings, learning was a by-product of what happened in the pre-round meeting, because a number of questions that arose had to be discussed. This study revealed that the discussions in the pre-round meetings were important for learning:

I regard this as a very important learning arena. In this arena questions are raised, and you do clinical evaluations and present several options for treatment. (D2)

The nurses stated they could learn much from discussions between doctors or between experienced nurses. They also noted that the information they learned could be transmitted both by nurses and doctors to one another, and this was something that had changed over time. Discussions with different healthcare professionals were highlighted as important for learning, as an experienced nurse expressed:

But also when the social worker comes up with questions we have not thought of, or experienced nurses discuss, and the doctor…, that is, we are equal [in our participation in the discussion], so you can learn from everyone in the group…. I experience it as a very important learning arena, because after all, you sit down and talk about something. (A4)

The results revealed that doctors and nurses may approach the same problem in different ways. One of the nurses mentioned the importance of the pre-round meetings in getting to know each other's ways of thinking to find the best solutions in practice:

You have to learn a little about how other work groups or other occupational groups think (A2)

Participants’ competence and experience influenced learning in the pre-round meeting. Inexperienced staff was most concerned with learning procedures related to diagnostics and treatment, but this changed, as described by a nurse with 30 years of experience:

I notice many differences among the new people [nurses]. Then [at this time in their career], their learning is very concrete; “we take that blood sample together with this”, and it is very tangible. “There should always be an X-ray before we do this, and operations tend to last that long, and they tend to lie in bed that long, and what do we have to take care of afterwards”? However, when they begin to gain more experience, the learning is more about exchanging experiences; you philosophize, are highly reflective together, and learn a lot because you have different skills. (A4)

However, it was also said that nurses and doctors have learning needs in common, such as changes, requiring updates by all colleges, for example, new guidelines, revised procedures, or organizational initiatives that affect everyday life in the hospital:

…. there are always new methods of treatments, new operations, and new types…, [new] ways of doing, shorter hospital stays… (A5)

Several participants argued that there is a connection between good collaboration and learning. One nurse emphasized that as part of this connection, it is important to know each other and develop confidence in one another. Another nurse mentioned that some doctors were very good at paying attention to nurses who are new to the unit, including them in learning opportunities, and explaining information. Then you learn more, and it is easier to ask questions.

### Factors Affecting Learning in Pre-Round Meetings

One of the nurses and two of the doctors noted that when consultants participate in the pre-round meeting, the learning outcomes improved. Both doctors and nurses further noted that the amount of time available for pre-round meetings also influenced learning outcomes. One of the nurses provided the following example:

Then they [the doctors] teach by drawing, depending on the amount of time available, of course. …… So, it's very instructive. (A6)

Participants mentioned that the learning outcomes of pre-round meetings are dependent on continuity of staff. When the staff changes from day to day, the potential for learning is negatively affected. Continuity of staff promotes increased possibilities for learning. When this is not the case, learning may be ignored, as described by one nurse in the orthopedic department:

…when you have new doctors every day, you have to start over again and again every day. You spend a lot of time (on starting over) that you might have used for simply learning. (C5)

It was said that when you have students on the unit, the focus on learning increases, and it is directed toward students’ learning:

… it (the pre-round meeting) is different when we have students on the unit from when we do not have them. When we have students, the pre-round meetings are always longer, more structured, and we spend more time teaching. They may be medical students or nursing students. (B1)

## Discussion

This study's aim was to gain a better understanding of the impact of pre-round meetings on the learning of nurses and doctors who attended them. Most of the nurses and doctors described the pre-round meeting as a learning arena with high potential; however, the results showed that various factors may influence the learning potential (Table 3). Our study stressed the importance of being aware of the potential for learning in the pre-round meeting, and recognizing this meeting as central to the clinical learning of healthcare professionals in their daily practice.

**Table 3.** Factors That Influence Learning in Pre-round Meetings.

Factors that increase learning Good collaboration Continuity of staff Consultant attending Students attending Interprofessional discussions Joint decision making Factors that inhibit learning Lack of structure Lack of time Lack of competence among attendees

### How may the Pre-Round Meeting Contribute to Clinical Learning?

Our study indicates that the direction of learning in the pre-round meeting is from both doctor-to-nurse and nurse-to-doctor. Based on our results, however, there is reason to believe there is asymmetry in the learning relationship. Although it emerged that even experienced consultants learned from pre-round meetings, it was clear the learning potential was greatest among the less experienced professionals. Staff with more experience tended to focus on exchanging experiences and reflecting on a higher level. Senior staff are role models, and for young nurses and doctors, role models are important for developing a holistic view of patients (Kleiven et al., 2016). Some participants mentioned the importance of the pre-round meetings as a learning arena for nursing and medical students. For students, it is important not only to learn their own subject areas, but also to learn about interprofessional collaboration (Hallin & Kiessling, 2016).

One of the study's main results was that the pre-round meeting facilitated learning by allowing staff from different professions to discuss issues concerning patients before the ward round. A discussion before the meeting with the patient can be useful for learning, and it has also been argued that learning occurs through making joint decisions in practice (Claridge, 2011, Radomski and Beckett 2011 cited in Bell et al., 2016). The participants reported achieving higher-level learning objectives, especially when learning occurred in discussions that arose at the intersection between their differences in approach and perspectives on common issues. Participants’ reports of learning from one another in pre-round meetings presupposes differences in their knowledge and competence that can complement each other during the learning process. This is the essence of interprofessional interactions (Bell et al., 2016).

Nisbet et al. (2015) argue that learning occurs in connection with seeing something from different perspectives. The discussion in the pre-round meeting often leads to a common understanding of an issue (Kyte et al., 2020). The results from the present study indicate that shared decision-making enhances learning in pre-round meetings to find solutions to clinical problems. Hence, an interprofessional approach is important for learning.

These findings can be understood in light of sociocultural learning theory (Wenger, 1998), which posits that learning is achieved through social processes (Wenger, 1998). In the pre-round meetings, the common goal of quality of care is fundamental (Kyte et al., 2020), and this interaction can lead to the establishment of communities of practice (Lave & Wenger, 1991; Wenger, 1998).

As said in the introduction, learning can be classified as formal, non-formal and informal (Commission of The European Communities, 2001). These three kinds of learning can be linked to various aspects of pre-round meetings. The nurses and doctors in our study perceived the pre-round meeting as a conscious learning arena, and one in which learning is a by-product. When learning is a by-product, it is perhaps more a product of interaction and discussion, and therefore, informal, yet important. Knowledge gained through interprofessional meetings may, in this way, contribute to care that is more comprehensive, which in turn, can enable a patient-centered focus (Kleiven et al., 2016; Nisbet et al., 2015). During interprofessional meetings, both individual and collective learning among healthcare professionals also contribute to improvements in patients (Nisbet et al., 2015).

Another important aspect of learning among the participants in our study was their understanding of how other professional groups think. This might be exactly what happens during interprofessional reflections. This finding is consistent with the conclusion of Nisbet et al. (2015), that communicating with someone from another profession is different from communicating with someone within one's own profession. When you meet, you learn to communicate interprofessionally, and you better understand the way other professionals think (Nisbet et al., 2015). Our study indicates that learning to understand each other is particularly important to achieve effective solutions in practice. Achieving consensus can also ensure better quality of care and treatment (Kyte et al., 2020).

Although learning can occur in informal contexts, it is often more successful when it is systematic and conducted in a prepared learning arena (Eraut, 2007; Stanley, 1998). This means that learning opportunities can be created by structuring “ward rounds” and including formal arenas for discussion in meetings before or after the visit (Stanley, 1998).

It can be questioned to what extent the pre-round meeting is an explicit learning arena, offering possibilities for more intended learning. However, when participants in the study referred to the pre-round meeting as a conscious learning arena, it implied possibilities for intended learning, corresponding with non-formal and perhaps formal learning sometimes.

Previous research indicates that if the learning process is made explicit, it provides a better environment for both individual and collective learning (Nisbet et al., 2015). A community of practices (Wenger, 1998) is also a learning arena, and it is important that everyone involved in the community is aware of this resource (Jaye et al., 2009). Awareness of opportunities in the pre-round meeting as a learning arena can help one extract more of the learning potential, both in terms of individual and collective learning, to achieve higher-levels learning objectives.

### The Importance of the Learning Environment in a Clinical Setting

The quality of the learning environment is important for success, which was revealed in our study. In practice, an environment conducive to learning is one in which learners feel safe and are treated with respect, which is consistent with the results of other studies (Aase et al., 2014; Adibelli & Korkmaz, 2017; Hallin & Kiessling, 2016; Nisbet et al., 2015). Units characterized by mutual respect among the staff use interprofessional arenas actively to promote collaboration and learning (Sjursen & Ytrehus, 2020). Students experience the staff in this type of department as role models, and role models are important for younger professionals (Aase et al., 2014; Kleiven et al., 2016). Hence, it is important to facilitate the use of explicit learning arenas where students are included. The pre-round meeting can be such a learning arena.

Cohn (2013) argues that hospital ward rounds must be given priority (Cohn, 2013). An important routine is to have a meeting before a ward round with patients (Royal College of Physicians & Royal College of Nursing, 2012; Willemann et al., 2006). Our study showed that several factors related to organization and structure can reduce the value of the pre-round meeting as a learning forum. Lack of time and continuity of staff were identified as such factors, consistent with other studies (Claridge, 2011; Laskaratos et al., 2015; Seltz et al., 2016). Lack of structure and competence were found to be challenges to implementation of pre-round meetings on several units (Kyte et al., 2020). Participants in our study reported that the pre-round meeting was more structured with a higher focus on learning when students were present and involved, and that the learning potential was greater when a consultant participated. This may be related to the consultant's higher level of competence, which enhanced the basis for decision-making and provided better structure (Kyte et al., 2020).

Our study showed that discussions involving members of different professions in pre-round meetings are important for learning. Pre-round meetings must be prioritized to use the learning potential effectively during this meeting. Factors such as time, continuity, competence, and structure are essential for the pre-round meeting to function as intended, and ultimately, to improve the quality of treatment and care (Kyte et al., 2020). When the pre-round meeting contributes to the quality of treatment and care, it may also be beneficial for learning. Hence, there is synergy between achieving quality treatment and care and utilizing the learning potential of pre-round meetings.

## Limitations

The limitations of our study should be mentioned. Data were obtained from a small number of departments in two hospitals in Norway. The results, are therefore, not necessarily representative of other types of wards and hospitals. The majority of nurses in the study were women and the majority of doctors were men, which does not necessarily reflect the gender distribution in Norwegian hospitals.

As nurses and doctors belong to professions that are always represented in pre-round meetings, we chose to interview them. However, if we had also interviewed other professionals participating in the pre-round meetings, we might have gained more knowledge about learning with a broader interprofessional perspective. It should also be noted that the choice of interviewing nurses and doctors separately and not in interprofessional groups, may have had an impact on the information gained through the interviews.

## Implications for Practice

The study findings provide new and useful knowledge about the educational value of pre-round meetings in clinical practice. By being aware of the opportunities the pre-round meeting has as a learning arena, healthcare professionals in their daily practice can extract more of the learning potential, both in terms of individual and collective learning. Therefore, health care professionals may raise the educational value of pre-round meetings by taking into consideration factors that may increase learning.

## Conclusion

The pre-round meeting is an arena with high learning potential, but various factors can influence this potential. The pre-round meeting allows professionals to discuss issues concerning patients before ward rounds. Learning takes place in discussions that arise when different professions with different perspectives on common issues meet. Both nurses and doctors emphasized that the pre-round meeting is a conscious learning arena and one where learning is a by-product. Some learning is concrete, while other learning occurs through the exchange of experiences and by philosophizing and reflecting together. An important aspect of learning among the participants was to understand how other professional groups think. By being aware of learning opportunities in the pre-round meeting, one can utilize more of its learning potential. A collaborative environment, continuity, competence, and availability of the staff and structured pre-round meetings are essential elements for achieving higher-level learning objectives. Further research should include allied health clinicians attending pre-round meetings, as for instance pharmacists, occupational therapists, physiotherapists, and social workers.
